# The Rolling of Food by Dung Beetles Affects the Oviposition of Competing Flies

**DOI:** 10.3390/insects9030092

**Published:** 2018-07-31

**Authors:** Manuel A. Ix-Balam, Maria G. A. Oliveira, Júlio Louzada, Jeremy N. McNeil, Eraldo Lima

**Affiliations:** 1 Departamento de Entomologia, Universidade Federal de Viçosa, Viçosa, MG 36570-900, Brazil; manuelbalam1786@gmail.com (M.A.I.-B.); malmeida@ufv.br (M.G.A.O.); eraldo.lima@ufv.br (E.L.); 2 Departamento de Biologia, Universidade Federal de Lavras, Lavras, MG 37200-000, Brazil; jlouzada@dbi.ufla.br; 3 Department of Biology, University of Western Ontario, London, ON N6A 5B7, Canada

**Keywords:** *Deltochilum furcatum*, dung beetle, interspecific competition, *Lucilia cuprina*, pygidial secretion, resource marking by both sexes

## Abstract

Flies are the main competitors of dung beetles for oviposition sites and rolling dung beetles relocate their food to reduce interspecific competition. Furthermore, dung beetles deposit chemical substances on the food ball that may repel fly larvae and certain predators. In the present study, using *Deltochilum furcatum*, a dung beetle that does not exhibit parental care and the blow-fly, *Lucilia cuprina*, we tested the hypothesis that pygidial secretions deposited on the food ball could also make it less attractive as an oviposition site for flies. Food balls rolled by either *D. furcatum* males or females received significantly fewer eggs that balls that had not been rolled by beetles. Also, flies laid significantly fewer eggs on food balls treated with secretions collected from male pygidial glands. Reduced fly oviposition may be a direct effect of compounds the beetles deposited, acting as an allomone, and/or an indirect negative effect on the microbial community that stimulates fly oviposition. A model of the reproductive biology of this species is proposed.

## 1. Introduction

Coprophagous beetles use dung and/or carrion as a food and nesting resource [1,2]. However, these nutrient-rich resources are ephemeral both in time and space, resulting in intense intra and interspecific competition for food [3]. Dipterans are the main interspecific competitors [2,3,4,5,6], so coprophagous beetles have evolved relocation strategies whereby resources are cut up and rolled to less accessible locations [1]. 

Favila [7] demonstrated that fresh male secretions, probably from the fifth abdominal sternite, were attractive to conspecific females, and secretions from different glands in *Canthon* species serve to deter competitors and predators [5,8], leading Cortez et al. [9] to suggest that these secretions may serve multiple purposes. This idea is supported by studies on other dung beetles: for example, male secretions from glands situated on the first abdominal sternite of *Kheper* species are involved in mating [10,11], while adults of *Oniticellus egregius* (Klug, 1855) produce secretions from the lateral edges the anterior abdominal segments that play a role in defence [12,13]. 

In the present study we carried out experiments to test the hypothesis that chemicals deposited during the rolling process by *Deltochilum furcatum* (Castelnau, 1840), a species strongly attracted to carrion [14], would reduce the ball’s attractiveness as an oviposition site for *Lucilia cuprina* (Wiedemann, 1830) (Diptera: Calliphoridae), as it also uses carrion as an oviposition site [15]. The secretions deposited by either sex during rolling reduced oviposition by *L. cuprina*, as did the mechanical application of male pygidial glands.

## 2. Materials and Methods

### 2.1. Biological Samples

The *D. furcatum* adults used in these experiments were collected using sardine baited pitfall traps set out in a fragment of a seasonal semideciduous Atlantic forest (20°45′22′′ S/42°51′44′′ W), near Viçosa, Minas Gerais State, Brazil. The beetles were separated by sex, held in 1000-mL plastic pots containing humid soil and fed fresh meat or sardine once a week. 

A colony of *L. cuprina* was established from maggots collected on meat left out in the field for several days on the campus of the Federal University of Viçosa. Adult flies were fed a mixture of honey and water, as well as fresh beef blood as the protein source for egg formation [15]. Pieces of meat placed in the cage served as oviposition sites and the eggs then transferred to 250-mL plastic containers (about 100 eggs/container) with milk powder diluted in water as a larval food source. Both species were reared at 27 ± 1 °C, 55% RH, 12L:12D. 

### 2.2. Behavioural Analysis

**Figure 1:**
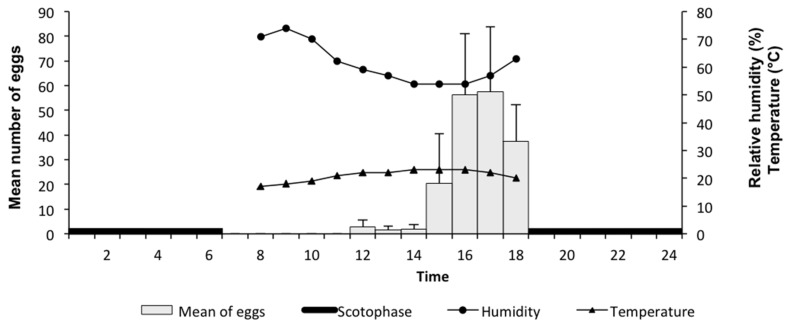
Mean (X ± SEM) number of eggs laid by *Lucilia cuprina* as a function of time during the photophase.

To determine the peak period of oviposition by *L. cuprina*, 10 pairs of 13 day-old flies were provided a 3-g ball (Ø = 19 mm) of fresh hamburger meat, similar in size and shape as those formed by beetles. The number of eggs were counted every hour throughout the photophase, as pretests showed that no eggs were laid during the scotophase. The experiment, performed at room temperature, had 16 replicates per hour (see Supplementary Materials File 1) (Figure 1). 

**Figure 2:**
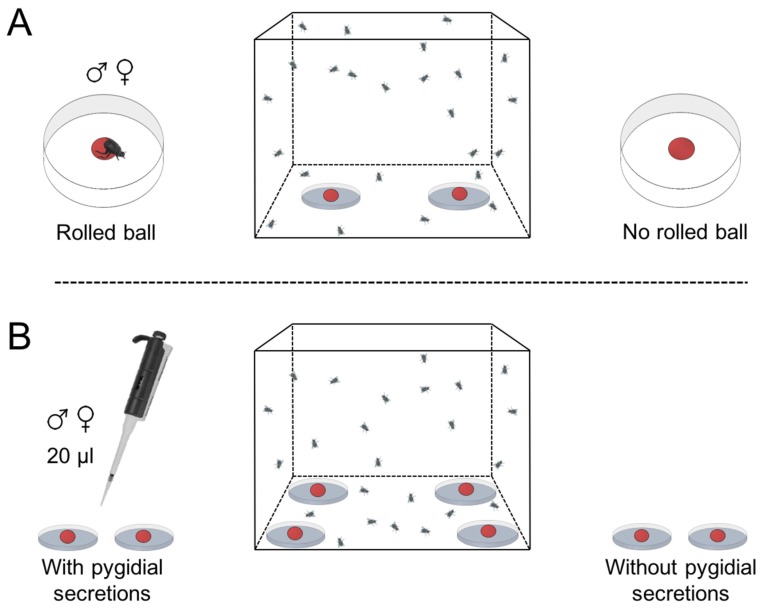
The experimental design to test the oviposition preference of *Lucilia cuprina* when there was a choice of (**A**) balls rolled by either male or female *Deltochilum furcatum* or (**B**) food balls impregnated with 20 μL of male and female pygidial secretions and untreated balls.

To determine the effect of *D. furcatum* rolling on subsequent fly oviposition, either a male or female beetle was randomly taken from the colony and left in a plastic arena with a meatball for 24 h. Control balls were held for 24 h under the same conditions but in the absence of a beetle. Subsequently, we placed a rolled and a control ball in a 0.5 m^3^ organza-covered cage during the last 4 h of photophase (peak oviposition period) with forty 13 ± 1 day old *L. cuprina* males and females (Figure 2A). At the end of each assay (six per sex) the total number of eggs on each ball was counted.

As observed with other Scarabaeinae species [8,12], *D. furcatum* releases pygidial secretions when disturbed. Therefore, male and female beetles were gently tapped and the secretions extruded collected under a stereoscope using sterilized glass capillary tubes (Micron capillaries glass-tube for micro-hematocrit without heparin). Samples of male and female secretions were held in 200 μL glass tubes at −6 °C until used in bioassays. The surface of two balls was treated with 20 μL of either male or female pygidial secretions and placed in an arena with two untreated balls. The attractiveness of treated and untreated balls as oviposition sites was determined using the same protocol described for beetle rolled vs. control balls above. There were three replicates for male and female secretions (Figure 2B).

### 2.3. Statistical Analyses

The effect of *D. furcatum* food rolling and pygidial secretions on the oviposition of *L. cuprina* were analyzed with a paired *t*-tests, using R software [16].

## 3. Results

**Figure 3:**
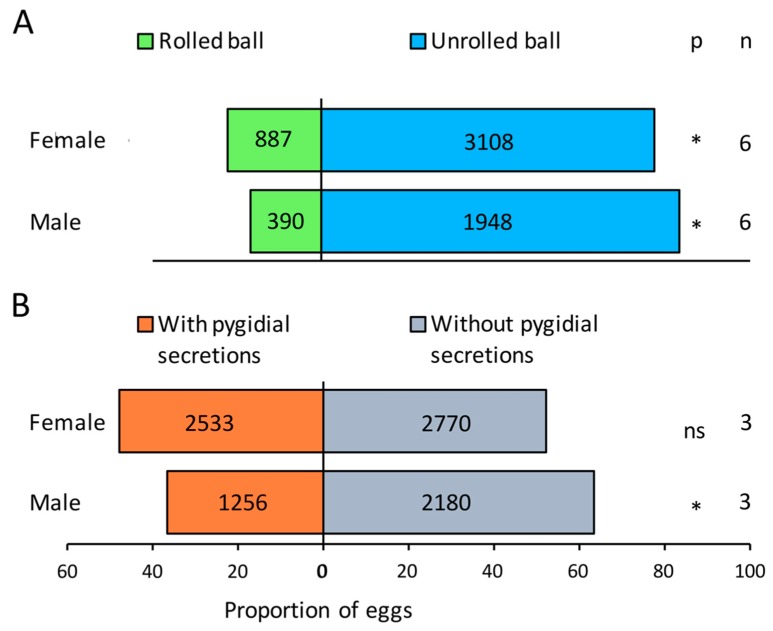
The relative proportion of eggs laid by *Lucilia cuprina* on (**A**) controlled food balls and balls rolled by either *Deltochilum furcatum* males or females, or (**B**) balls treated with pygidial secretions from field collected *D. furcatum* males or females. * = *p* < 0.05; ns = no significant difference. The values inside the bars indicate the total number of *L. cuprina* eggs.

The mean number of *L. cuprina* eggs laid during the 4 h experiment comparing unrolled balls and those rolled by beetles was 389.7 ± 146.3 for male and 665.8 ± 82.2 for female *D. furcatum* treatments (See Supplementary Materials File 2). Significantly fewer eggs were laid on rolled balls than control balls, regardless of which sex did the rolling (Figure 3A).

In the experiment comparing unrolled balls and those treated with either male and female extracts, the mean number of eggs laid was 1145.3 ± 566.5 and 1767.7 ± 720.2 for male and female treatments, respectively (see Supplementary Materials File 3). Significantly fewer eggs were laid in balls treated with pygidial secretions from males but not of females (Figure 3B). 

## 4. Discussion

Both male and female *D. furcatum* adults deposit chemicals on the ball that limit oviposition by *L. cuprina*. This is very different from the situation reported for *Canthon cyanellus cyanellus* (LeConte, 1859), where only balls marked by males are less attractive to *Calliphora* larvae in a choice assay [5]. Obviously the two studies were testing the choice behaviors of different life history stages of the same species, but it seems rather unlikely that this would be the cause of the difference observed in the two studies. We believe that the marked differences in the exploitation of resources between the two beetle species would explain the difference in marking behavior.

In the case of *C. cyanellus cyanellus* only males mark the ball, but both sexes collaborate in ball rolling and parental care of the brood, which is located in a chamber containing several food balls [5,17]. In contrast, *D. furcatum* buries one ball at a time and exhibit no parental care. Thus, in the absence of either pair formation or brood care, the most effective means of reducing interspecific competition would be when both sexes marked.

**Figure 4:**
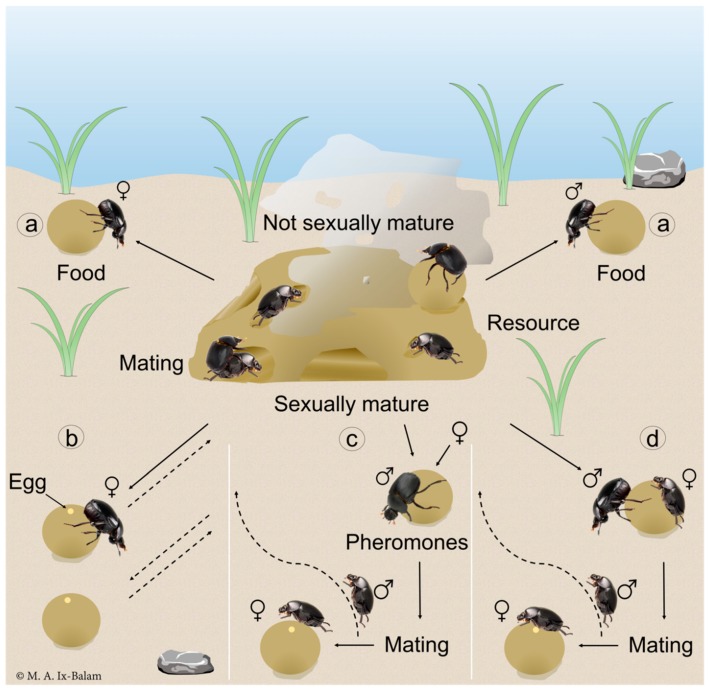
Possible scenarios for mating and resource exploitation by both male and female *Deltochilum furcatum* that would explain why both sexes mark food balls.

Based our findings, together with observations of adults in a large arena, we believe there are several reproductive scenarios for *D. furcatum*, that would explain why both sexes produce secretions during the rolling of food that reduce interspecific competition (Figure 4). Adults of both sexes are attracted to the carcass, where immature beetles independently prepare and feed on food balls, while sexually mature individuals could also mate (Figure 4 scenario a). A female mating on the carcass may then independently roll a ball for some distance, oviposit an egg and bury the ball before returning to repeat the process; this she could do repeatedly without a male as she would have an adequate supply of sperm (Figure 4 scenario b). A mature male could prepare a ball, roll it some distance from the carcass and then release sex pheromones to attract a virgin female (Figure 4 scenario c). Following mating, the male returns to the food source and repeat the process to locate additional mates, while the mated female oviposits, buries the ball and then returns to the food source to continue as in scenario b.

There are two other possible variations: where a female sits on a ball being rolled by a male but does not mark or help in any way, or when a female is rolling a ball and a male joins her (Figure 4 scenario d). The sex rolling the ball usually buries it, mating may occur, the female oviposits, the sexes separate, and continue independently to repeat any one of the three scenarios described. In all cases, the balls would be susceptible to attack by dipterans, so in the absence of parental care or true collaboration between adults, as with *D. furcatum*, marking by both sexes would certainly play an important role in reducing interspecific competition.

While balls rolled by either sex reduced oviposition, only male pygidial secretions of showed activity when applied mechanically. There are a number of possible alternate explanations for the difference between the experiments using balls rolled by adult females beetles and those just treated with secretions only. First, the pygidial secretions were collected from adults of unknown age/sexual maturity and these factors are known to affect gland content in dung beetles [2,5,7,18,19]. Second, the samples were extracted by disturbing the beetles rather than through normal production in the presence of a food ball. Third, the time and mode of application involved in deposition of the secretions by beetles is obviously more complex and longer that for manual application using a syringe. These factors, individually or in combination, could affect the outcome of the different assays and merit further attention. Also, there are a number of exocrine glands located on the abdomens of *Deltochilum* species [18], which in *Canthon* sp. are known to serve several functions [8,9], so it is possible that secretions from several different glands may be applied during rolling.

The secretions deposited may act as allomones, directly affecting female fly oviposition behavior. However, during rolling the food may be contaminated with fungi and bacteria that negatively affect the beetle larvae [20] and chemical substances from abdominal exocrine glands help inhibit the growth of these microorganisms [21], including the pygidial glands [9]. Several fly species have been shown to preferentially lay eggs on substrates with bacteria rather than on sterile ones [22,23,24,25,26] as the bacteria may be essential dietary components for developing maggots [25,27] and/or may cause the release essential nutrients from the substrate though enzymatic action [25,28]. Furthermore, female *L. cuprina* uses volatile compounds released by bacteria developing on the food source when selecting oviposition sites [22]. Thus, it is possible that the beetle secretions modify the microbial community in a way that reduces the suitability of the resource and *L. cuprina* females avoid oviposition sites that would be suboptimal for the development of their progeny.

## 5. Conclusions

The data presented certainly support the hypothesis that glandular secretions placed on the ball by both sexes of *D. furcatum* during rolling reduces oviposition by *L. cuprina* and could reduce interspecific competition. However, considerably more research is required to (i) determine the origin; (ii) the composition of the beetle secretions (preliminary analyses of glands suggest lipids and carbohydrates) that deter oviposition; (iii) elucidate persistence of the compounds and the changes that render the resource less attractive as an oviposition site for flies; and (iv) conduct experiments to quantify the benefits accrued from reduced interspecific competition.

## Supplementary Materials

The following raw data are available online at http://www.mdpi.com/2075-4450/9/3/92/s1. The number of eggs deposited by *Lucilia cuprina* (i) as a function of time during the photophase (File 1); (ii) on balls rolled by *Deltochilum furcatum* and control balls (File 2); and (iii) balls with and without *Deltochilum furcatum* pygidial secretions (File 3).

## References

[1] Halffter G., Edmonds D.W. (1982). The Nesting Behavior of Dung Beetles (Scarabeinae): An Ecological and Evolutive Approach.

[2] Scholtz C.H., Davis A.L.V., Kryger U. (2009). Evolutionary Biology and Conservation of Dung Beetles.

[3] Hanski I., Cambefort Y., Hanski I., Cambefort Y. (1991). Competition in dung beetles. Dung Beetles Ecology.

[4] Bornemissza G.F. (1970). Insectary studies on the control of dung breeding flies by the activity of the dung beetle, *Onthophagus gazella* F. (Coleoptera: Scarabaeinae). Aust. Entomol..

[5] Bellés X., Favila M.E. (1983). Protection chimique du nid chez *Canthon cyanellus cyanellus* LeConte (Col. Scarabaeidae). Bull. Soc. Entomol. Fr..

[6] Hirschberger P., Degro H.N. (1996). Oviposition of the dung beetle *Aphodius ater* in relation to the abundance of yellow dungfly larvae (*Scatophaga stercoraria*). Ecol. Entomol..

[7] Favila M.E. (1988). Chemical labelling of the food ball during rolling by males of the subsocial coleopteran *Canthon cyanellus cyanellus* Leconte (Scarabaeidae). Insectes Soc..

[8] Cortez V., Favila M.E., Verdú J.R., Ortiz A.J. (2012). Behavioral and antennal electrophysiological responses of a predator ant to the pygidial gland secretions of two species of Neotropical dung roller beetles. Chemoecology.

[9] Cortez V., Verdú J.R., Ortiz A.J., Trigos Á.R., Favila M.E. (2015). Chemical diversity and potential biological functions of the pygidial gland secretions in two species of Neotropical dung roller beetles. Chemoecology.

[10] Burger B.V., Petersen W.G.B. (2002). Semiochemicals of the Scarabaenae: VI. Identification of EAD-active constituents of abdominal secretion of male dung beetle, *Kheper nigroaeneus*. J. Chem. Ecol..

[11] Burger B.V., Petersen W.G.B., Weber W.G., Munro Z.M. (2002). Semiochemicals of the Scarabaeinae. VII: Identification and synthesis of EAD-active constituents of abdominal sex attracting secretion of the male dung beetle, *Kheper subaeneus*. J. Chem. Ecol..

[12] Burger B.V., Petersen W.G.B., Tribe G.D. (1995). Semiochemicals of the Scarabaeinae, V: Characterization of the defensive secretion of the dung beetle *Oniticellus egregius*. Z. Naturforsch. C.

[13] Burger B.V., Mucignat-Caretta C. (2014). First investigation of the semiochemistry of South African dung beetle species. Neurobiology of Chemical Communication.

[14] Falqueto S.A., Vaz-De-Mello F., Schoereder J.H. (2005). Are fungivorous Scarabaeidae less specialist?. Ecol. Austral.

[15] Capinera J.L., Capinera J.L. (2008). Australian sheep blowfly, *Lucilia cuprina* Wiedemann (Diptera: Calliphoridae). Encyclopedia of Entomology.

[16] R Development Core Team (2014). The R Foundation for Statistical Computing.

[17] Favila M.E. (2001). Historia de vida y comportamiento de un escarabajo necrofago: *Canthon cyanellus cyanellus* LeConte (Coleoptera: Scarabaeinae). Folia Entomol. Mex..

[18] Pluot-Sigwalt D. (1995). La diversité du système des glandes tégumentaires abdominales des scarabaeidae (S. Str.) (Coleoptera): Morphologie et répartition des structures cuticulaires. Ann. Soc. Entomol. Fr..

[19] Houston W.W.K. (1986). Exocrine glands in the forelegs of dung beetles in the genus *Onitis* F. (Coleoptera: Scarabaeidae). Aust. Entomol..

[20] Favila M.E. (1993). Some ecological factors affecting the life-style of *Canthon cyanellus cyanellus* (Coleoptera Scarabaeidae): An experimental approach. Ethol. Ecol. Evol..

[21] Cortes-Gallardo V., Favila M.E., Estrada V.E., Equihua M.A., Luna L.C., Rosas A.J. (2007). Actividad antifúngica del ácido 4-metoxi fenilácetico producido en las glándulas esternales de machos del escarabajo rodador *Canthon cyanellus cyanellus* (Coleoptera: Scarabaeinae). Entomología Mexicana.

[22] Emmens R.L., Murray M.D. (1983). Bacterial odours as oviposition stimulants for *Lucilia cuprina* (Wiedemann) (Diptera: Calliphoridae), the Australian sheep blowfly. Bull. Entomol. Res..

[23] Romero A., Broce A., Zurek L. (2006). Role of bacteria in the oviposition behaviour and larval development of stable flies. Med. Vet. Entomol..

[24] Lam K., Babor D., Duthie B., Babor E.M., Moore M., Gries G. (2007). Proliferating bacterial symbionts on house fly eggs affect oviposition behaviour of adult flies. Anim. Behav..

[25] Thompson C.R., Brogan R.S., Scheifele L.Z., Rivers D.B. (2013). Bacterial interactions with necrophagous flies. Ann. Entomol. Soc. Am..

[26] Zheng L., Crippen T., Holmes L., Singh B., Pimsler M., Benbow M., Tarone A., Dowd S., Yu Z., Vanlaerhoven S. (2013). Bacteria mediate oviposition by the black soldier fly, *Hermetia illucens* (L.), (Diptera: Stratiomyidae). Sci. Rep..

[27] Lam K., Geisreiter C., Gries G. (2009). Ovipositing female house flies provision offspring larvae with bacterial food. Entomol. Exp. Appl..

[28] Bing X., Attardo G.M., Vigneron A., Aksoy E., Scolari F., Malacrida A., Weiss B.L., Aksoy S. (2017). Unravelling the relationship between the tsetse fly and its obligate symbiont *Wigglesworthia*: Transcriptomic and metabolomic landscapes reveal highly integrated physiological networks. Proc. R. Soc. B.

